# Deficiency of X-linked *TENT5D* causes male infertility by disrupting the mRNA stability during spermatogenesis

**DOI:** 10.1038/s41421-021-00369-9

**Published:** 2022-03-08

**Authors:** Jiangshan Cong, Yihong Yang, Xin Wang, Ying Shen, Hui-Tao Qi, Chunyu Liu, Shuyan Tang, Sixian Wu, Shixiong Tian, Yiling Zhou, Xiaojin He, Lingbo Wang, Mo-Fang Liu, Feng Zhang

**Affiliations:** 1 Obstetrics and Gynecology Hospital, NHC Key Laboratory of Reproduction Regulation (Shanghai Institute for Biomedical and Pharmaceutical Technologies), State Key Laboratory of Genetic Engineering, Institute of Reproduction and Development, Fudan University, Shanghai, China; 2 Shanghai Key Laboratory of Female Reproductive Endocrine Related Diseases, Shanghai, China; 3 Key Laboratory of Obstetric, Gynecologic and Pediatric Diseases and Birth Defects of Ministry of Education, West China Second University Hospital, Sichuan University, Chengdu, Sichuan China; 4 Center of Reproductive Medicine, Sichuan University, Chengdu, Sichuan China; 5 School of Life Science, Hangzhou Institute for Advanced Study, University of Chinese Academy of Sciences, Hangzhou, Zhejiang China; 6 State Key Laboratory of Molecular Biology, Shanghai Key Laboratory of Molecular Andrology, CAS Center for Excellence in Molecular Cell Science, Shanghai Institute of Biochemistry and Cell Biology, Chinese Academy of Sciences-University of Chinese Academy of Sciences, Shanghai, China; 7 Reproductive Medicine Center, Department of Obstetrics and Gynecology, The First Affiliated Hospital of Anhui Medical University, Hefei, Anhui China; 8 NHC Key Laboratory of Study on Abnormal Gametes and Reproductive Tract, Anhui Medical University, Hefei, Anhui China; 9 Key Laboratory of Population Health Across Life Cycle, Anhui Medical University, Ministry of Education of the People’s Republic of China, Hefei, Anhui China; 10 School of Life Science and Technology, Shanghai Tech University, Shanghai, China

**Keywords:** Gene expression profiling, Mechanisms of disease, Transcriptomics

Dear Editor,

Infertility has become a worldwide health problem affecting ~10% of couples^1^. However, genetic causes of male infertility remain largely elusive and unexplained. In addition, advances in assisted reproduction technologies (ART) to obtain biological children are in increasing demand.

Oligoasthenoteratozoospermia (OAT) is a common type of male infertility with genetic heterogeneity, manifesting low sperm concentrations, reduced motility and various malformations. A group of OAT-affected men fail to father a biological child via conventional ART, such as intracytoplasmic sperm injection (ICSI), probably due to severe defects in spermatogenesis. Modeling human genetic variants in mice has been shown to be efficient in establishing gene–disease relationships for male infertility^2^. In addition, mouse models have superiority for the exploration and optimization of ART approaches. Herein, by taking advantage of the availability of OAT patients and a gene-edited mouse model, we investigated a novel genetic cause of male infertility and tested a “FACS + ROSI” (fluorescence activated cell sorting + round spermatid injection) strategy for a potential compensatory ART approach.

First, whole-exome sequencing (WES) and bioinformatic analyses were performed to analyze our cohort of 186 unrelated Han Chinese men with OAT. A hemizygous stop-gain variant (c.637G > T [p.Glu213*]) of X-linked *TENT5D* (NCBI GenBank: NM_001170574.2) was identified in the proband (II-1 in Fig. 1a) from nonconsanguineous family HX001. Sanger sequencing showed that the mother (I-2 in Fig. 1a) is a heterozygous carrier. The variant p.Glu213* with glutamicacid that located at a conserved position of TENT5D changed to a stop codon was predicted to be deleterious by MutationTaster and CADD tools (Fig. 1a; Supplementary Table S1). This *TENT5D* variant is novel and absent in human population genome databases, including the 1000 Genomes Project and gnomAD (Supplementary Table S1). In the OAT patient harboring a hemizygous *TENT5D* variant, the sperm DNA fragmentation index and high DNA stainability were dramatically increased (Supplementary Table S2). The spermatozoa from the hemizygous *TENT5D*-mutated patient displayed multiple heads and/or multiple flagella upon hematoxylin and eosin (H&E) staining and scanning electron microscopy (SEM) detection (Fig. 1b; Supplementary Fig. S1 and Table S2). Transmission electron microscopy (TEM) also revealed multiple heads and/or multiple flagellar ultrastructural abnormalities (Fig. 1c). These findings suggest that *TENT5D* deficiency may cause human male infertility with OAT.

**Fig. 1:**
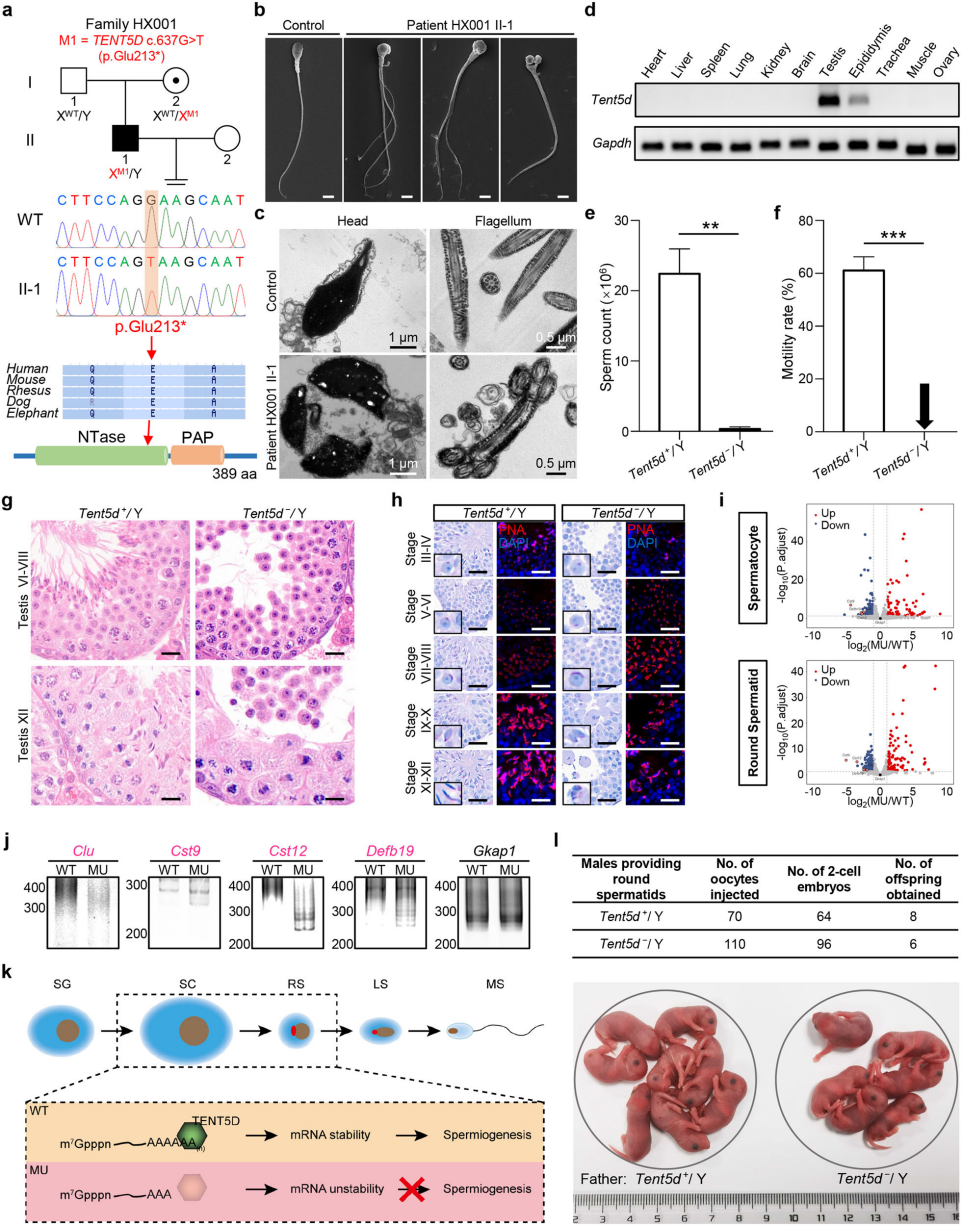
Deficiency of X-linked *TENT5D* causes male infertility by disrupting mRNA stability during spermatogenesis. **a** Pedigree of family HX001 affected by a stop-gain *TENT5D* variant. The black-filled square indicates the male case with oligoasthenoteratozoospermia. Sanger sequencing confirmed the hemizygous *TENT5D* variant in proband HX001 II-1. Schematic representation of TENT5D and phylogenetic conservation of the variant location. **b**, **c** SEM (**b**) and TEM (**c**) analyses of the spermatozoa from a male control individual and the *TENT5D*-associated proband. Scale bars, 5 μm. **d** Expression of *Tent5d* was investigated by reverse-transcription PCR in various tissues from adult male mice. *Gapdh* was used as an internal control. **e**, **f** The sperm count (**e**) and percentages of motile sperm (**f**) in WT (*Tent5d*^+^/Y) and *Tent5d*-mutated (*Tent5d*^−^/Y) male mice (**P < 0.01, ***P < 0.001). Error bars represent the standard error of the mean. Two-tailed Student’s paired or unpaired *t*-tests were used as appropriate. **g** H&E staining performed on testis sections of *Tent5d*^+^/Y and *Tent5d*^−^/Y male mice. Scale bars, 20 μm. **h** PAS staining and immunostaining assays were performed on adult mouse testis sections. Scale bars, 20 μm. **i** Volcano plots of the transcriptome analysis showing the expression changes of mRNAs in spermatocytes and round spermatids of MU and WT male mice. **j** PCR-based poly(A) test of *Clu*, *Cst9*, *Cst12*, *Defb19* and *Gkap1* mRNAs in the testes of MU and WT male mice. The downregulated genes were marked in pink. **k** Schematic model showing that TENT5D stabilizes mRNAs by extending the length of poly(A) tails in spermatocytes and round spermatids. **l** Representative development of mouse embryos. The offspring was obtained by “FACS + ROSI” using round spermatids from *Tent5d*^+^/Y and *Tent5d*^−^/Y male mice.

Considering that TENT5D is highly conserved between humans and mice (83.8% similarity in consensus positions), we used a mouse model to investigate whether TENT5D deficiency plays a causative role in male infertility. Murine ortholog X-linked *Tent5d* is preferentially expressed in the testis and epididymis (Fig. 1d). Furthermore, *Tent5d* was highly expressed at 3 to 4 weeks, the time point matching the first wave of spermatocytes producing spermatids in wild-type (WT, *Tent5d*^+^/Y) testes (Supplementary Fig. S2).

To further investigate the physiological role of *Tent5d* during spermatogenesis, we generated *Tent5d*-mutated mice using CRISPR/Cas9 technology and tentatively ruled out potential off-target effects of the gRNA on a set of genomic regions that we predicted based on sequence similarity (Supplementary Table S3). Finally, we obtained *Tent5d*-mutated (MU, *Tent5d*^−^/Y) male mice by introducing a frameshift deletion (c.622_625del), which was predicted to cause premature translational termination (Supplementary Fig. S3). Furthermore, the expressions of *Tent5d* and TENT5D were almost absent in the testes of *Tent5d*-mutated male mice (Supplementary Fig. S4). We also found that *Tent5d*-mutated male mice were completely sterile (Supplementary Fig. S5a).

Eight-week-old *Tent5d*-mutated male mice exhibited dramatically smaller testes as compared with WT controls (Supplementary Fig. S5b). Sperm concentration and motility were significantly reduced in *Tent5d*-mutated male mice (Fig. 1e, f). The testicular lumen of 8-week-old WT male mice was composed of spermatogenic cells at various stages. In contrast, the testicular lumens of age-matched *Tent5d*-mutated male mice were full of round spermatids but lacked elongating spermatids and later forms (Fig. 1g). These “round spermatid-like” and few elongated sperms were also found in both caput and cauda epididymides of *Tent5d*-mutated male mice (Supplementary Fig. S6a).

To further investigate the role of TENT5D during spermatogenesis, we performed periodic acid-Schiff (PAS) staining and immunostaining assays using peanut agglutinin fluorescent dye. Proacrosomal granules began to form until late-round spermatid cells appeared abnormal (Fig. 1h; Supplementary Fig. S6b). TEM analysis also indicated that spermatids did not develop normally to the acrosome phase in *Tent5d*-mutated adult male mice (Supplementary Fig. S6c). We also observed that multinucleated giant cells (symplasts) contained multiple spermatids within a single plasma membrane in the testicular lumen of *Tent5d*-mutated adult male mice (Supplementary Fig. S7). There were few symplasts in the testes of the 4-week-old *Tent5d*-mutated male mice (Supplementary Fig. S8a). However, the symplasts were obviously increased in the 8-week-old *Tent5d*-mutated male mice (Supplementary Fig. S8b), indicating that the symplasts appeared in late spermatogenesis.

In germ cells, intercellular bridges, which are produced during mitosis and meiosis to connect the daughter cells, are thought to be important for intracellular communication^3,4^. F-actin (a marker of cell junctions) and TEX14 (a marker of intercellular bridges) exhibited abnormal localization in the testicular lumen of *Tent5d*-mutated male mice (Supplementary Fig. S9a, b). Moreover, the TUNEL assay showed a high level of apoptosis in the testicular lumen of 8-week-old *Tent5d*-mutated male mice, while no apoptosis signals were observed in the 4-week-old group (Supplementary Fig. S9c). Collectively, these observations indicate that the appearance of spermatogenic abnormalities at step 8 (late-round spermatids) of spermiogenesis in *Tent5d*-mutated male mice might be due to abnormal connections between cells.

TENT5D belongs to the TENT5 subfamily and is expected to have noncanonical poly(A) polymerase activities and roles in mRNA stability^5^. To test this hypothesis, we first examined the catalytic properties and nucleotide selection of TENT5D. In vitro tailing assays showed that TENT5D extended 5′ [^32^P]-labeled A_15_ only in the presence of ATP and the mixture but not UTP, CTP or GTP (Supplementary Fig. S10), indicating that TENT5D is an active noncanonical poly(A) polymerase. Next, we explored the role of TENT5D on mRNA stability during mouse spermatogenesis. Considering the fact that there is active transcription in spermatocytes (SC) and round spermatids (RS)^6^, we isolated SC and RS from the testes of *Tent5d*-mutated and WT mice (Supplementary Fig. S11a, b) and performed transcriptome analyses using RNA sequencing. The qualities of isolated germ cells and sequencing were characterized by hierarchical clustering (Supplementary Fig. S11c). This analysis identified obviously more downregulated genes than upregulated genes in *Tent5d*-mutated germ cells (|*FC*| > 2, *P.adjust* < 0.05; Fig. 1i; Supplementary Fig. S11d, Tables S4 and S5). For example, *Clu*, *Cst9*, *Cst12* and *Defb19* were downregulated in both *Tent5d*-mutated SC and RS, with *Gkap1* as a negative control (Supplementary Fig. S12a, b). These data indicate that TENT5D tends to stabilize mRNAs during spermatogenesis. Gene Ontology (GO) analysis of the downregulated genes revealed multiple distinct gene clusters (Supplementary Fig. S12c). Importantly, in line with defective spermatogenesis and male infertility in *Tent5d*-mutated male mice, the affected genes were enriched under the GO terms of reproduction in multicellular organisms, germ cell development, spermatid differentiation, single fertilization and fertilization, implying that TENT5D regulates multiple cellular programs in male germ cells. Notably, cell adhesion-related factors *Adam26a* and *Cldn34c4* were downregulated in SC and RS, respectively, indicating that *Tent5d* deficiency might also affect cell adhesion during mouse spermatogenesis (Supplementary Fig. S13a). Taken together, these data support that TENT5D is a noncanonical poly(A) polymerase important for the stability of mRNAs during spermatogenesis.

Poly(A) tail length is a key determinant of mRNA turnover, and mRNAs with shortened poly(A) tails are easily degraded^7^. Using real-time quantitative PCR assays, we confirmed the downregulation of *Adam26a*, *Cldn34c4*, *Clu*, *Cst9*, *Cst12* and *Defb19* in mouse testes, with *Gkap1* as the negative control (Supplementary Figs. S12a and S13b). By performing the poly(A) test assay, we found that each downregulated gene had shorter poly(A) tails in the testes of *Tent5d*-mutated male mice than in those of WT controls, while there were no significant changes for the *Gkap1* control group (Fig. 1j; Supplementary Fig. S13c). Collectively, these results reveal that *Tent5d* deficiency could shorten some mRNA poly(A) tails and further result in mRNA instability, thereby affecting the process of spermatogenesis (Fig. 1k). It has been reported that the length of poly(A) tails can also influence mRNA translational efficiency in maturing oocytes and early embryos^8^, and we cannot exclude the possibility that poly(A) tail shortening in *Tent5d*-mutated male germ cells might affect translational efficiency and subsequently contribute to germ cell defects.

ICSI using ejaculated sperm is an effective treatment for severe OAT^9^. Here, the OAT case HX001 II-1, harboring a hemizygous *TENT5D* stop-gain variant, also received ICSI treatment in clinic. Unfortunately, the HX001 couples revealed a poor ICSI outcome (Supplementary Table S6). Similarly, ICSI for *Tent5d*-mutated male mice was also inefficient, limiting its potential application in overcoming *TENT5D*-associated male infertility (Supplementary Fig. S14).

To develop an efficient method for rescuing *TENT5D*-associated sterility, we tested the “FACS + ROSI” strategy in the *Tent5d*-mutated mouse model. To guarantee precise isolation of haploid spermatids from testicular mixtures, we employed FACS to enrich round spermatids and then injected them into oocytes obtained from WT female mice (Supplementary Fig. S15a). Two-cell embryos were successfully obtained. After embryo transfer, we obtained six live offspring at half of the efficiency of the controls (Fig. 1l). Also, the genotypes of offspring fit the expected patterns (Supplementary Fig. S15b). Therefore, “FACS + ROSI” provides a possible approach to enable patients with *TENT5D*-associated OAT to have their own genetic offspring.

In summary, our genetic and functional data based on human and mouse experimental evidence strongly suggest that hemizygous stop-gain mutations of *TENT5D*/*Tent5d* as a novel genetic cause of primary male infertility with OAT and provide insights into the molecular mechanism by which *TENT5D* function in the process of spermatogenesis. Although *TENT5D* deficiency may account for a small fraction of the OAT cases, our findings are consistent with the high genetic heterogeneity of male infertility. Furthermore, the success of the “FACS + ROSI” approach in *Tent5d*-mutated male mice indicates an effective treatment for human male infertility caused by *TENT5D* mutations. This study will provide new information for genetic counseling and clinical guidance of male infertility with OAT.

## Supplementary information

Supplementary table S5

Supplementary Information

Supplementary table S4
