# Supplementary material for: Deficiency of X-linked TENT5D causes male infertility by disrupting the mRNA stability during spermatogenesis
Source: Cell Discov. 2022 Mar 8;8:23. doi: 10.1038/s41421-021-00369-9 (PMC8901658; doi:10.1038/s41421-021-00369-9)
Supplement: Supplementary file 2 — Supplementary Information [file 41421_2021_369_MOESM2_ESM.pdf]

Supplementary information

Materials and methods

Human patients

A cohort of 186 Chinese men affected by oligoasthenoteratozoospermia was recruited from the West China Second University Hospital of Sichuan University and the First Affiliated Hospital of Anhui Medical University. The clinical phenotypes of all patients are isolated infertility without any primary ciliary dyskinesia-associated symptoms (such as bronchitis, pneumonia, sinusitis, or otitis media). The study was approved by the institutional review boards at Fudan University, West China Second University Hospital of Sichuan University, and the First Affiliated Hospital of Anhui Medical University. Informed consent was obtained from each patient in this study.

Whole-exome sequencing (WES) and bioinformatics analysis

Genomic DNAs were extracted from peripheral blood samples of human patients using the DNeasy Blood and Tissue Kit (QIAGEN, 51106). WES and bioinformatics analysis were performed as described previously[1,2]. Briefly, genome DNA was processed using the Agilent SureSelect Human All Exon V6 Kit or Twist Bioscience's Human Core Exome Kit before the DNA sequencing using HiSeq 2000 or NovaSeq 6000 platform (Illumina). Raw data were aligned to the human genome reference assembly (GRCh37/hg19) using the Burrows-Wheeler Aligner software[3], and PCR duplicates were removed with Picard

software. We then used the ANNOVAR software for functional annotation with a variety of genome datasets and bioinformatic tools, including OMIM, Gene Ontology, SIFT, PolyPhen-2, MutationTaster, 1000 Genomes Project and gnomAD[4-8]. Sanger sequencing was conducted to validate the candidate pathogenic variants using the PCR primers listed in Supplementary Table S7.

Semen parameters analysis

Semen samples of human individuals were collected through masturbation after 2 - 7 days of sexual abstinence and examined in the source laboratories during routine biological examination according the World Health Organization (WHO) guidelines. Sperm morphology was assessed by hematoxylin and eosin (H&E) staining and electron microscopy assays. We examined at least 200 spermatozoa to evaluate the percentages of morphologically abnormal spermatozoa.

For mouse semen samples, we collected them from the cauda epididymides. Then we diluted these samples in 1 mL of human tubal fluid (HTF; Millipore, Cat. # MR-070-D) before incubation at 37℃ for 15 minutes. Sperm counts and motility were analyzed by a computer-assisted semen analysis system.

Sperm chromatin structure assay

DNA fragmentation index (DFI) and high DNA stainability (HDS) were measured by flow cytometry using sperm DNA fragmentation staining kit (ANKEBIO). At least 5000 sperm

cells were measured and repeated twice for each subject. The acridine orange staining solution was added to stain the sites of single-strand DNA breaks red and the double-strand DNA breaks green. Then the red and green fluorescence signals were collected by flow cytometry, and the DFI and HDS values were calculated using the Flowjo software.

Mouse model

Tent5d-mutated mice were generated using CRISPR-Cas9 technology. Cas9 and sgRNA were prepared as described previously[9]. Founder mice were crossed to WT C57BL/6 mice to establish the mutant mouse line[1,2,10]. The founder mice and their offspring were identified by Sanger sequencing with the primers listed in Supplementary Table S7. Young mice (4-week-old) and adult mice (8-week-old) were used in this study. All animal experiments were carried out in accordance with the recommendations of the Guide for the Care and Use of Laboratory Animals of the US National Institutes of Health. The study was approved by the animal ethics committee at the School of Life Sciences of Fudan University.

Reverse-transcription PCR (RT-PCR) and real-time quantitative PCR (RT-qPCR)

For RT-PCR, total RNAs were extracted from mouse various tissues using RNeasy Mini Kit (Qiagen). Approximately 1 μg of RNA was reversely transcribed to cDNA using SuperScript Ⅲ Reverse Transcriptase (Invitrogen) according to the manufacturer's instructions. RT-PCR was performed with cDNA, and *Gapdh* was used as an internal

control (Supplementary Table S7).

For RT-qPCR, total RNAs were extracted from human spermatozoa and mouse testes and reversely transcribed as described above. The cDNAs were diluted 5-fold to be used as templates for RT-qPCR with AceQ qPCR SYBR Green Master Mix (Vazyme). The results were analyzed and shown as relative mRNA levels of the CT (cycle threshold) values, which were then converted as fold changes. *GAPDH*/*Gapdh* were used as internal controls, and primers for RT-qPCR are presented in Supplementary Table S7.

Electron microscopy assays

For scanning electron microscopy (SEM), human sperm specimens were subjected to fixation in 2.5% glutaraldehyde, washed with 0.1 mol/L phosphate buffer for 30 minutes and postfixed in 1% osmic acid. Next, the samples were progressive dehydration with ethanol and dried with a CO_2 critical-point dryer (Eiko HCO-2, Hitachi). Subsequently, the samples were mounted on aluminum stubs, sputter-coated by an ionic sprayer meter (Eiko E-1020, Hitachi) and analyzed via SEM (Stereoscan 260) under an accelerating voltage of 20 kV.

For transmission electron microscopy (TEM), human semen and mouse testis/epididymis were fixed in 2.5% glutaraldehyde. The samples were progressively dehydrated with ethanol gradient (50%, 70%, 90%, and 100%) and 100% acetone. Next, the samples were embedded in Epon 812, sliced by an ultra-microtome and stained with uranyl acetate and lead citrate. Finally, the samples were observed and photographed by

TEM (TECNAI-10, Philips) with an accelerating voltage of 80 kV.

Immunofluorescence staining

The fresh mouse testes were dissected and fixed in 4% paraformaldehyde at 4℃ for 24 hours. The samples were progressively embedded in OCT compound. Then the cryosections of mouse testes were washed with phosphate-buffered saline (PBS) and blocking in 10% donkey serum for 1 hour at room temperature. The slides were subsequently incubated with the following primary antibodies overnight at 4℃: anti-TENT5D (PA5-31591, Thermo, 1:100), peanut agglutinin (PNA, VECTOR, 1:100), F-actin (P1951, Sigma, 1:1000). Then we wash the slides with PBS containing 0.1% (v/v) Tween20, incubated with Cy3-conjugated AffiniPure goat anti-rabbit IgG (111-165-003, Jackson, 1:4000) secondary antibodies for 1 hour at room temperature. Finally, the slides were counterstained with 4,6-diamidino-2-phenylindole (DAPI, Sigma Aldrich) and photographed with a laser scanning confocal microscope (Zeiss LSM 880).

Histological analysis of mouse tissues

Fresh mouse testis and epididymis tissues were fixed in Bouin's solution and 4% paraformaldehyde, respectively. After 24 hours at 4℃, the samples were embedded in paraffin and sectioned at 5 μm intervals by a microtome. The sections were routinely stained with H&E and periodic acid-Schiff (PAS) reagent, respectively.

Immunohistochemistry

Fresh mouse testis tissues were fixed in 4% paraformaldehyde at 4℃ for 24 hours and embedded in paraffin. The sections samples were performed by a microtome at 5 μm. Then, the samples were incubated with anti-TEX14 (18351-1-AP, Proteintech, 1:100).

TdT-mediated dUTP nick-end labeling

Fresh mouse testis tissues were fixed in 4% paraformaldehyde at 4℃ for 24 hours. We then embedded the samples in optimal cutting temperature compound. Tissue sections were prepared and mounted on glass slides. Apoptotic analysis was performed using the TMR Tunel Cell Apoptosis Detection Kit (G1502, Servicebio).

***In vitro* tailing assay**

RNA substrate 5′-AAAAAAAAAAAAAAA-3′ were synthesized in genscript. After 48 hours transfection with Myc-TENT5D, HEK 293T cells were harvested. Cells were lysed in Buffer A (150 mM NaCl, 50 mM Tris-HCl [pH 7.0], 5 mM EDTA, 0.1% Triton X-100). Myc-TENT5D was immunopurified with Anti-c-Myc antibody (Sigma, 3956) incubated with Protein A beads or Protein G beads (Sigma) and washed 6 times, with 3 times Buffer A, following 3 times high salt buffer A (500 mM NaCl). 5′end [γ-^{32}P]-ATP (PerkinElmer) labeled RNA substrates were incubated with beads in the presence of 40 U/μL RNasin (Promega) in the reaction buffer (25 mM Tris-HCl [pH 7.0], 50 mM KCl, 0.02 mM EDTA, 10 mM $MgCl_2$, 10 mM $MnCl_2$, 0.2 mM DTT and 1 mM each NTPs or mix) at 37℃ for 60

minutes. Samples were run on 15% polyacrylamide sequencing gel with 7M urea. Gel image was obtained using phosphor imaging plate (Fujifilm) and read by FLA 9000 (Fujifilm).

PCR-based poly(A) test assay

PAT assays were performed as described previously with specific primers (Supplementary Table S8)[11]. In brief, total RNAs extracted from testis were incubated with p[dT]$_{12-18}$, oligo[dT]-anchor, and T4 DNA ligase before reverse transcription reaction for generating cDNA. The polyadenylation state of specific mRNAs was analyzed by PCR using a mRNA gene-specific primer (F), oligo(dT)-anchor (R). The specific mRNAs were analyzed by PCR using a mRNA gene-specific primer (F), mRNA gene-specific poly(A) start primer (R). The PCR reaction consisted of initial denaturation of 93℃ for 5 minutes; then 30 cycles of 93℃ for 30 seconds, 57℃ for 1 minute, and 72℃ for 1 minute; followed by a final extension of 72℃ for 7 minutes. The amplified DNAs were separated on 5% polyacrylamide TBE gel, and stained with ethidium bromide.

Isolation of spermatogenic cells from mouse testes

Mouse spermatocytes and round spermatids were isolated from mouse testes as previously described[12]. In brief, total spermatogenic cells were extracted from the seminiferous tubules of male mice, stained by Hoechst 33342 (Acros Organics), and then separated through fluorescence activated cell sorting (FACS). The isolated spermatogenic cells were

further confirmed by their distinct nuclear morphology (Hoechst 33342 staining of nuclei).

Bulk RNA-seq data processing and analysis

Transcriptomes in spermatocytes and round spermatids were sequenced using the Illumina sequencing platform, with three biological replicates in this assay. Sequencing raw data were detected using the FastQC v.11.8 (https://www.bioinformatics.babraham.ac.uk/projects/fastqc/). Adapters were trimmed off with the Trimmomatic program (v.0.36)[13]. PCR duplicates were removed as previously reported[14]. FASTX_Toolkit (v.0.0.14) was used to eliminate poor-quality bases, discard low-quality and short reads, and trim 9 bases from the 5′ end. After filtering, the paired reads were aligned with the mouse genome (GRCm38/mm10, release-100, Ensembl) using the STAR software (v.2.7.0a)[15]. The deepTools (v.3.2.1) was used to get the coverage track (*.bw) from the normally mapped reads (*.out.bam) for visualization on IGV browser (v.2.10.0)[16,17]. The HTSeq software (v.0.6.0) was adopted to generate the count matrix[18].

Differential expression analysis

R software packages were used for differentially expressed genes (DEGs) analysis with the counting matrix. The differentially expressed mRNA levels were analyzed using DESeq2[19]. The shrinkage approach of DESeq2 was used to implement a regularized logarithm (rlog) transformation for PCA analysis (*prcomp* function). The median-of-ratios method of DESeq2 was used to calculate the normalization counts. Only the transcripts with $|FC| \geq 2$

and *padj* (*P.adjust*) < 0.05 were considered as DEGs. Clustering analysis of DEGs was conducted with numeric matrix transformed from normalization counts using the *ComplexHeatmap* package[20]. Volcano plots were assembled using the DESeq2 transformed output (*log2FC* and *-log10padj*) and plotted using the *ggplot2* package. The target gene enrichment test of downregulated genes was annotated based on biological processes Gene Ontology terms using *clusterProfiler* package[21].

Intracytoplasmic sperm injection and intracytoplasmic round spermatid injection in mice

Superovulation and intracytoplasmic sperm injection in mice were performed as previously described[1,10]. For intracytoplasmic round spermatid injection, the haploid round spermatids were obtained from wild-type and *Tent5d*-mutated adult male mice using FACS as described above. Oocytes were collected from superovulated 8-week-old B6D2F1 (C57BL/6×DBA2) female mice, pre-activated by 10 mM $SrCl_2$ for 0.5 hour. Then, the sorted haploid round spermatids were injected into pre-activated oocytes with a piezo-driven pipette. The injected oocytes were further activated using $SrCl_2$ for 3.5 hours, and then transferred into KSOM medium at 37℃ under 5% CO_2 in air for 20 hours to reach two-cell embryos. Two-cell embryos were transferred into the oviducts of pseudopregnant ICR females at 0.5 days post coitum.

Supplementary Figures

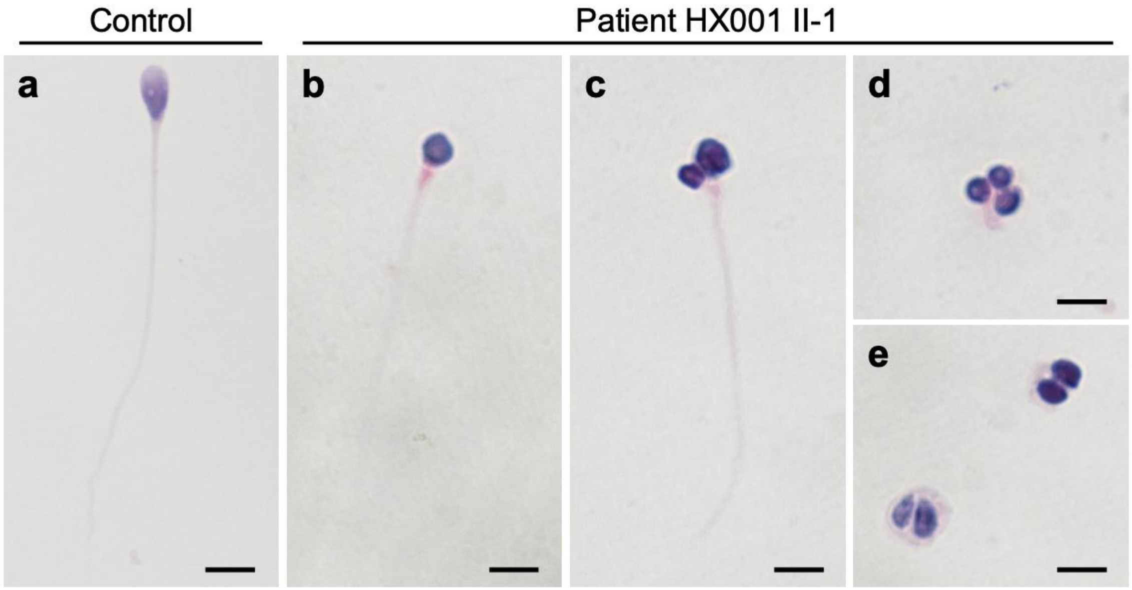

Supplementary Fig. S1 H&E staining of the spermatozoa from a fertile control man and the infertile male harboring a hemizygous *TENT5D* stop-gain variant.

a Normal morphology of the spermatozoa from a fertile control man. **b-e** Most spermatozoa obtained from the male patient (HX001 II-1) harboring a hemizygous *TENT5D* stop-gain variant displayed multiple heads and/or multiple flagella. Scale bars: 10 μm.

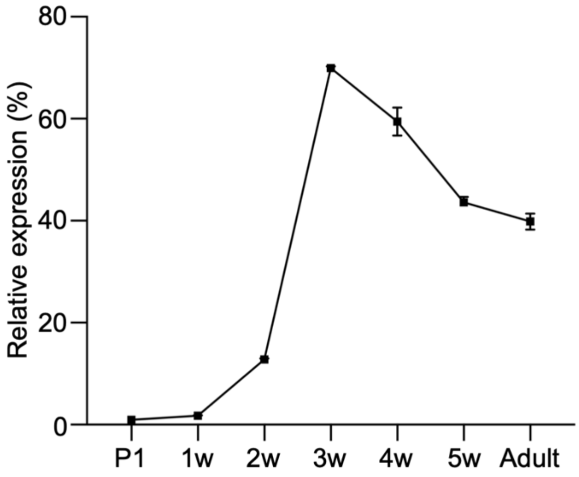

Supplementary Fig. S2   The expression levels of *Tent5d* mRNA in the testes of wild-type mice at different ages.

Investigations on relative expression levels of *Tent5d* in mouse testes from postnatal day 1 (P1) to 5-week-old (5w) and adult age using RT-qPCR. *Gapdh* was used as an internal control.

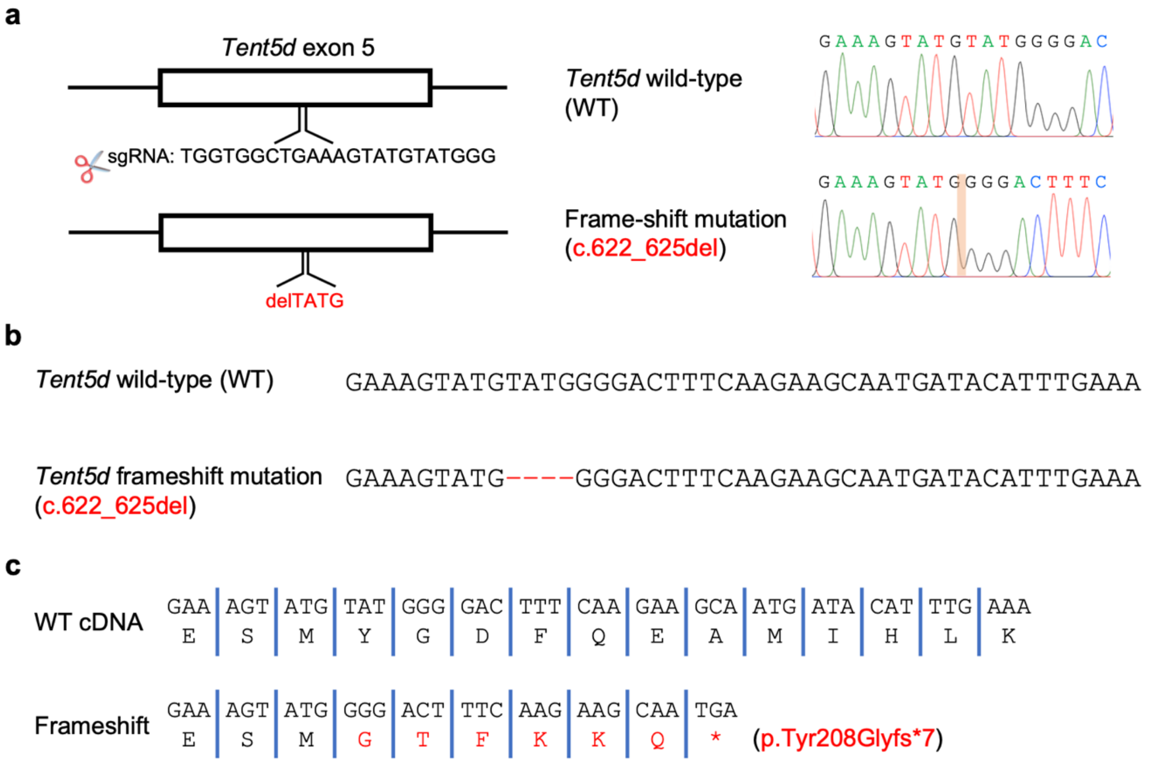

Supplementary Fig. S3 Generation of frameshift mutations in mouse *Tent5d* using CRISPR-Cas9 technology.

a Schematic illustration of the targeting strategy for generating *Tent5d*-mutated mice. The gRNA targeted exon 5 of mouse *Tent5d*. The rejoining junction after 4-bp deletion (c.622_625del) in the *Tent5d*-mutated sequence was highlighted. **b** The *Tent5d* frameshift mutation (c.622_625del) was generated in mouse *Tent5d* using CRISPR-Cas9 technology. The corresponding mutated nucleotides were shown in red. **c** The frameshift mutation was predicted to cause premature translational termination (p.Tyr208Glyfs*7) of mouse *Tent5d*. The frameshift-mutated amino acid sequences were shown in red.

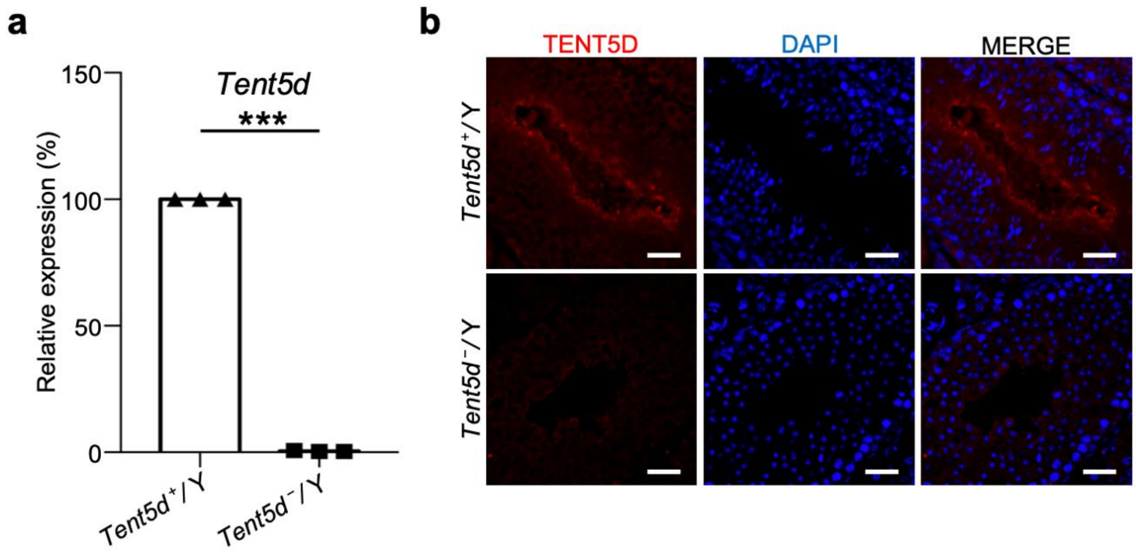

Supplementary Fig. S4 Expression analysis of *Tent5d* mRNA and protein in the *Tent5d*-mutated male mice.

a RT-qPCR assay showed that the level of *Tent5d* mRNA was almost undetectable in the testis from *Tent5d*-mutated (*Tent5d*$^{-}$/Y) male mice. The data represent the means ± standard deviations (SDs) of three independent experiments. Two-tailed Student's paired or unpaired *t* tests were used as appropriate (***$p < 0.001$). **b** Expression and location of TENT5D (red) protein in mouse testis revealed by immunofluorescence staining assay. DNA was counterstained with DAPI as a nuclei marker. Scale bars: 40 μm.

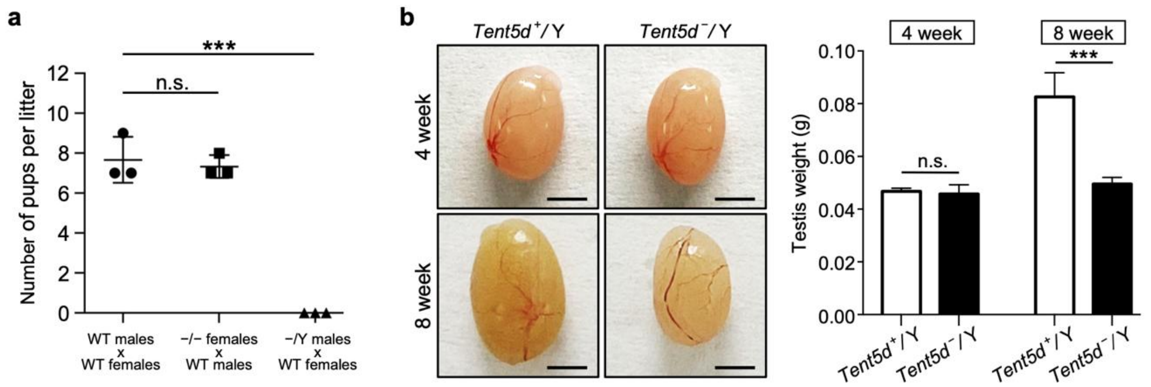

Supplementary Fig. S5 Deficiency of *Tent5d* leads to sterility in male mice.

a Fertility of wild-type mice and *Tent5d*-mutated mice. *Tent5d*-mutated male mice were completely sterile (***$p < 0.001$), whereas homozygous *Tent5d*-mutated female mice were fertile (n.s.: not significant). **b** Testis sizes and weights in WT (*Tent5d* $^{+}$/Y) and *Tent5d*-mutated (*Tent5d* $^{-}$/Y) male mice at the ages of 4 weeks and 8 weeks. Scale bars: 2 mm.

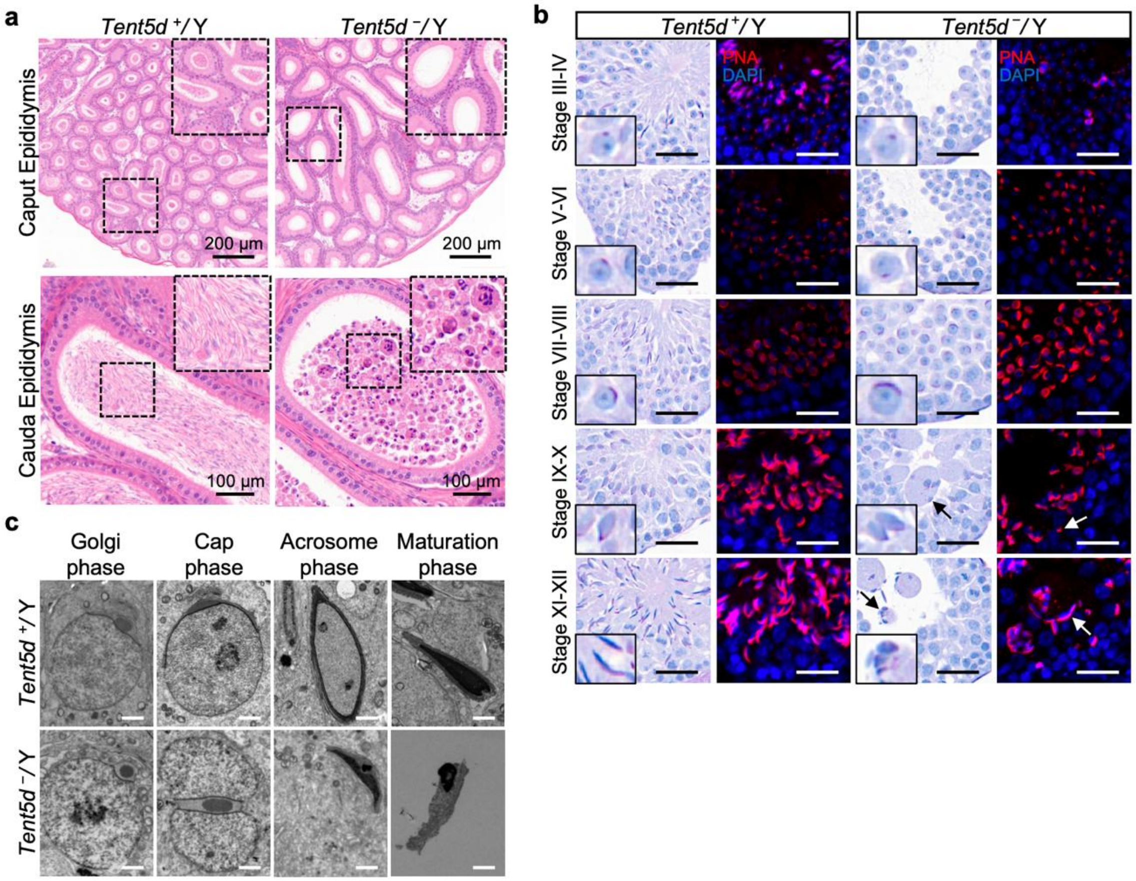

Supplementary Fig. S6 Phenotypic analyses of *Tent5d*-mutated (*Tent5d* $^{-}$/Y) male mice.

a H&E staining of caput and cauda epididymis cross sections of wild-type (*Tent5d* $^{+}$/Y) and *Tent5d*-mutated (*Tent5d* $^{-}$/Y) adult male mice. Spermatozoa are found in the caput and cauda epididymides of *Tent5d* $^{+}$/Y male mice, but the counts of spermatozoa are significantly decreased in the *Tent5d* $^{-}$/Y adult male mice. **b** The enlarged images of Fig. 1h. PAS staining and immunostaining assays were performed on the testis sections from adult male mice. The arrows point to the abnormal morphologies of proacrosomal granules. Scale bars: 20 μm. **c** TEM analysis of spermatids in ultrathin testis sections from *Tent5d* $^{+}$/Y and *Tent5d* $^{-}$/Y adult male mice. Compared with normal acrosomal development in the *Tent5d*$^{+}$/Y male mice, abnormal acrosomal morphologies occur at the cap stage in *Tent5d* $^{-}$/Y male mice. Scale bars: 2 μm.

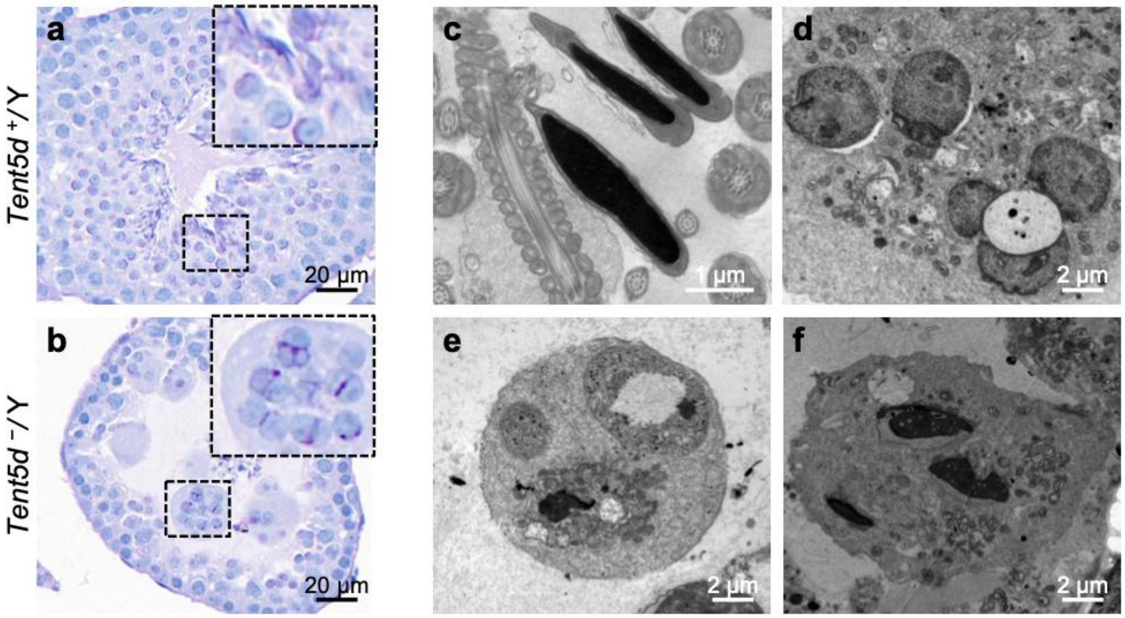

Supplementary Fig. S7 The formation of symplasts in the *Tent5d*-mutated (*Tent5d*$^{-}$/Y) male mice.

a, b Testis histology of wild-type (*Tent5d*$^{+}$/Y) (**a**) and *Tent5d*$^{-}$/Y adult male mice (**b**) by PAS staining. The seminiferous epithelium has a normal appearance in *Tent5d*$^{+}$/Y male mice, and the symplasts of round spermatids are present in *Tent5d*$^{-}$/Y adult male mice. **c-f** TEM assays were performed on the cauda epididymis from *Tent5d*$^{+}$/Y (**c**) and *Tent5d*$^{-}$/Y adult male mice (**d-f**). The symplasts are present in *Tent5d*$^{-}$/Y adult male mice.

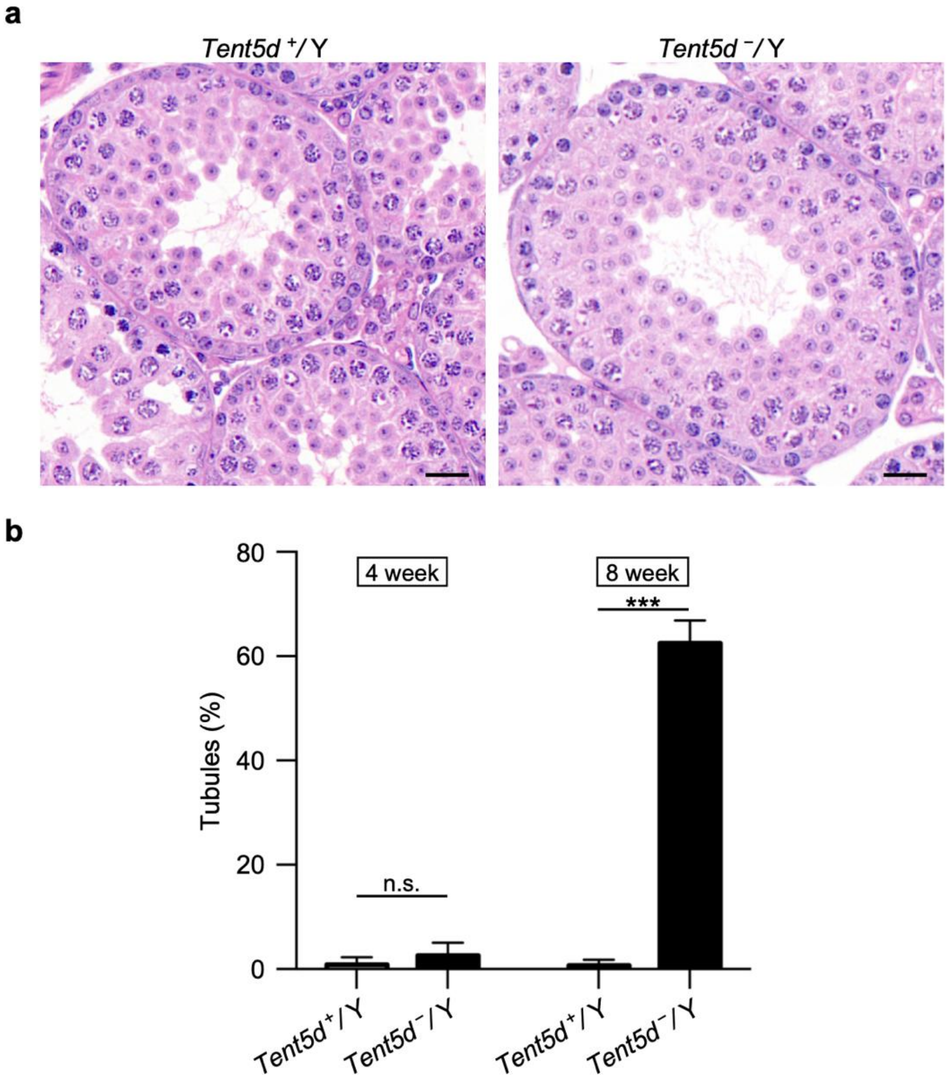

Supplementary Fig. S8 No symplasts was detected in the testes of *Tent5d*-mutated (*Tent5d*$^{-}$/Y) male mice at 4 weeks old.

a H&E staining of testis cross sections of wild-type (*Tent5d*$^{+}$/Y) and *Tent5d*$^{-}$/Y male mice of 4 weeks old. Scale bars: 20 μm. **b** Quantification of seminiferous tubules containing symplasts in *Tent5d*$^{+}$/Y and *Tent5d*$^{-}$/Y male mice at ages of 4 weeks and 8 weeks (n.s.: not significant, ***P < 0.001).

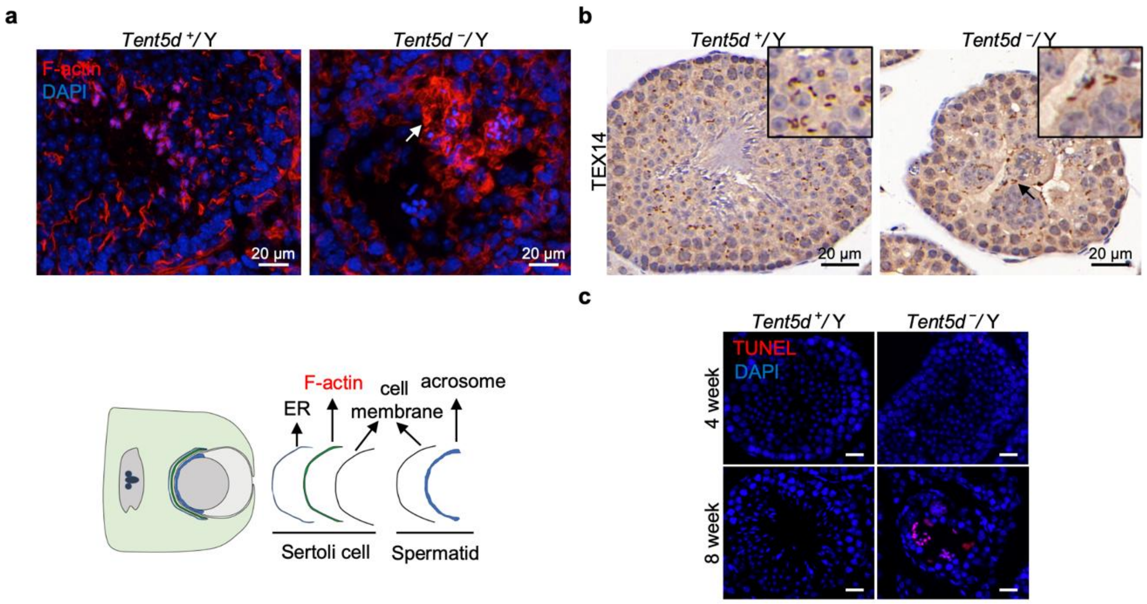

Supplementary Fig. S9 Affected F-actin and TEX14 immunostaining in the testes of *Tent5d*-mutated (*Tent5d*$^{-}$/Y) male mice.

a Immunofluorescence staining assays were performed on the testis sections of wild-type (*Tent5d*$^{+}$/Y) and *Tent5d*-mutated (*Tent5d*$^{-}$/Y) male mice using F-actin (red) fluorescent dye. DNA was counterstained with DAPI (blue) as a nuclear marker. The white arrow points to the abnormal localization of F-actin. The schematic diagram shows the positional relationship between F-actin and acrosome in the testis of *Tent5d*$^{+}$/Y male mice. **b** Immunohistochemical images showing the localization of TEX14 at intercellular bridges on the seminiferous tubules of *Tent5d*$^{+}$/Y and *Tent5d*$^{-}$/Y male mice. The arrow points to the abnormal localization of TEX14. **c** TUNEL assays of the testis sections from wild-type and *Tent5d*-mutated male mice (top: 4-week-old, and bottom: 8-week-old). Scale bars: 20 μm.

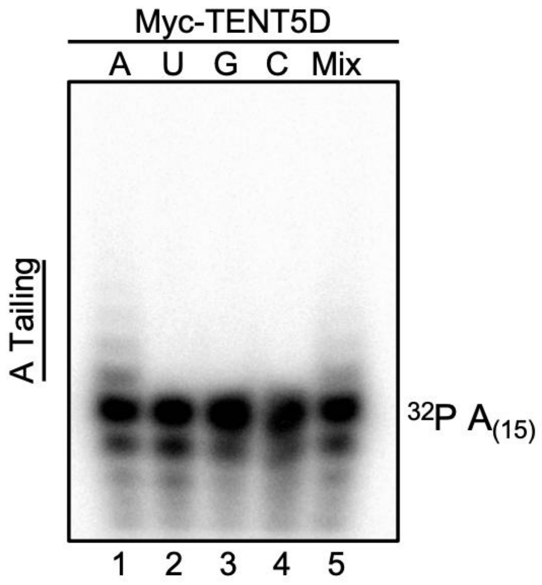

Supplementary Fig. S10 TENT5D is an active noncanonical poly(A) polymerase.
In vitro tailing assay shows that TENT5D extends 5′ [^{32}P]-labeled A_{15} substrate only in the presence of ATP.

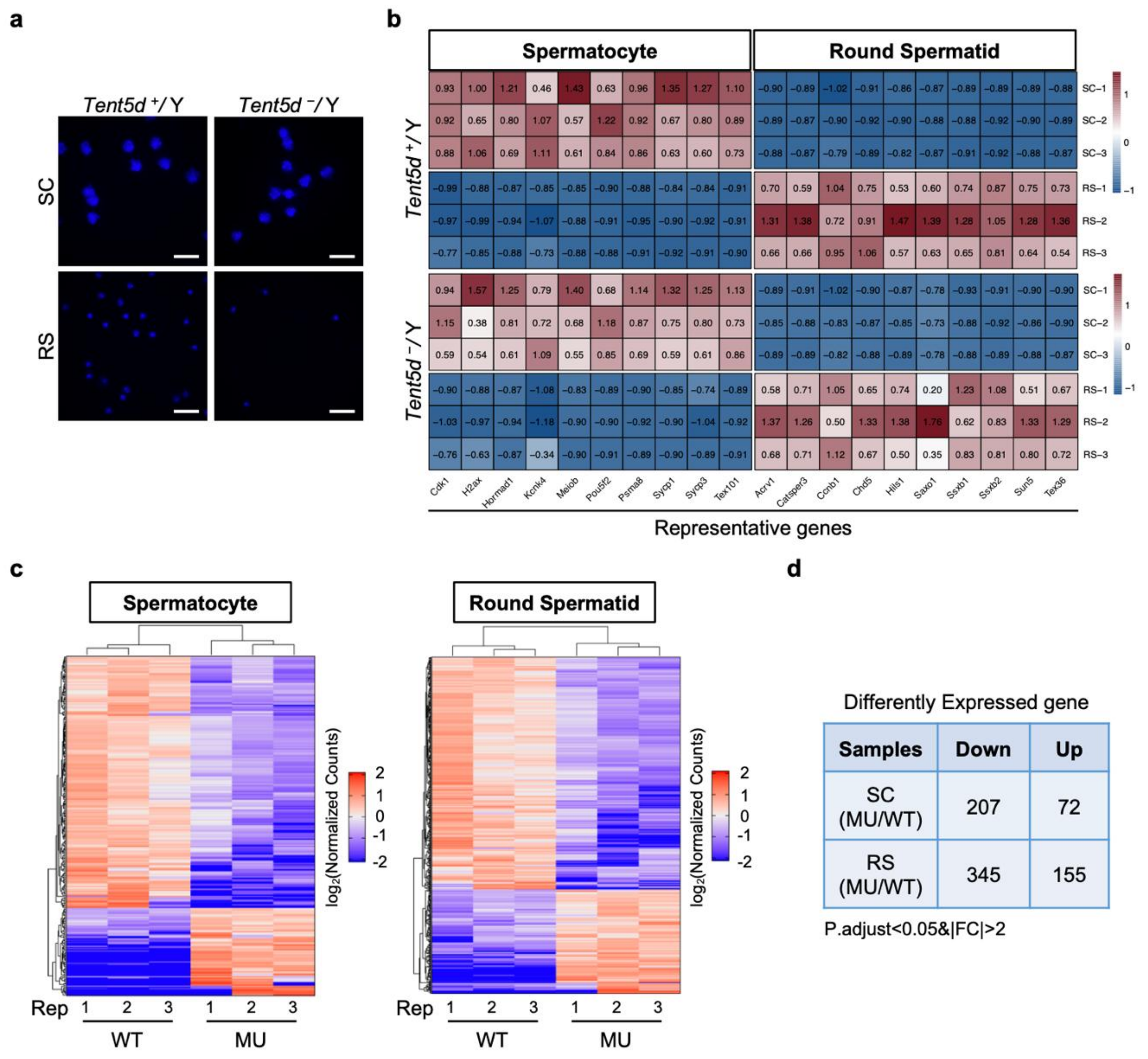

Supplementary Fig. S11 Quality control and sequencing analysis of isolated germ cells.

a Morphological confirmation of spermatocytes (SC) and round spermatids (RS) isolated from the testes of wild-type (WT, *Tent5d*$^{+}$/Y) and *Tent5d*-mutated (MU, *Tent5d*$^{-}$/Y) male mice. Scale bars: 50 μm. **b** Transcriptome analysis of representative genes of SC and RS isolated from the testes of *Tent5d*$^{+}$/Y and *Tent5d*$^{-}$/Y male mice. **c** Hierarchical clustering of significantly differently expressed mRNAs in the spermatocytes and round spermatids between WT and MU male mice. The expression levels of mRNAs were represented by a color scale. “Blue” represents the low relative expression level, and “red” represents the high relative expression level. Each column represents a biological repeated sample, and each row represents a single mRNA. **d** The numbers of differentially expressed genes between *Tent5d*-mutated groups and WT controls (|*FC*|>2, *P.adjust*<0.05) in SC and RS.

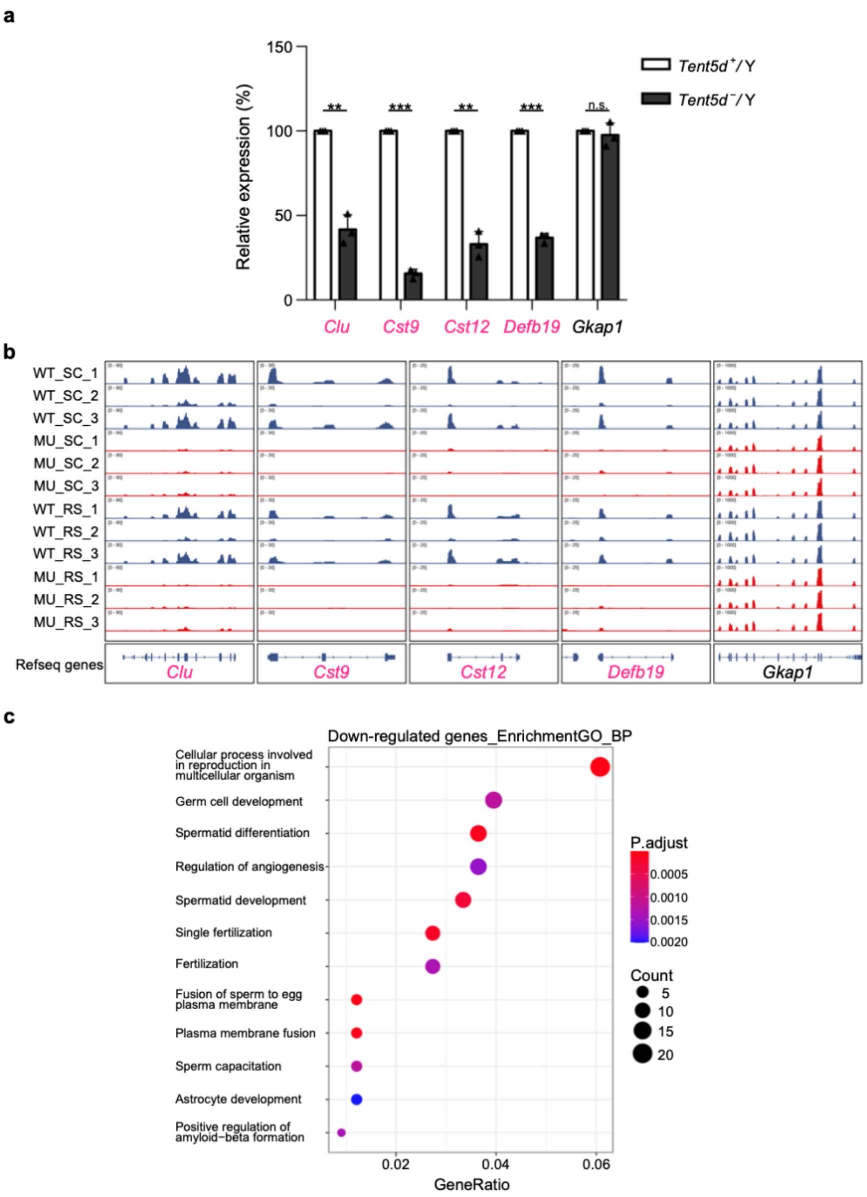

Supplementary Fig. S12 The stability of mRNAs is dysregulated in germ cells of *Tent5d*-mutated male mice.

a RT-qPCR analyses of *Clu*, *Cst9*, *Cst12*, *Defb19* and *Gkap1* mRNA expressions in the testis of *Tent5d*-mutated (MU, *Tent5d*$^{-}$/Y) and wild-type (WT, *Tent5d*$^{+}$/Y) male mice, with *Gapdh* serving as a normalized reference (**P < 0.01, ***P < 0.001, n.s.: not significant). The downregulated genes were marked in pink. **b** Genome browser snapshots of transcriptome-seq showing the mRNAs of mouse *Clu*, *Cst9*, *Cst12* and *Defb19* were downregulated in the germ cells of *Tent5d*-mutated male mice, with *Gkap1* as a negative control. The downregulated genes were marked in pink. **c** Gene ontology analysis of the downregulated genes in *Tent5d*-mutated groups.

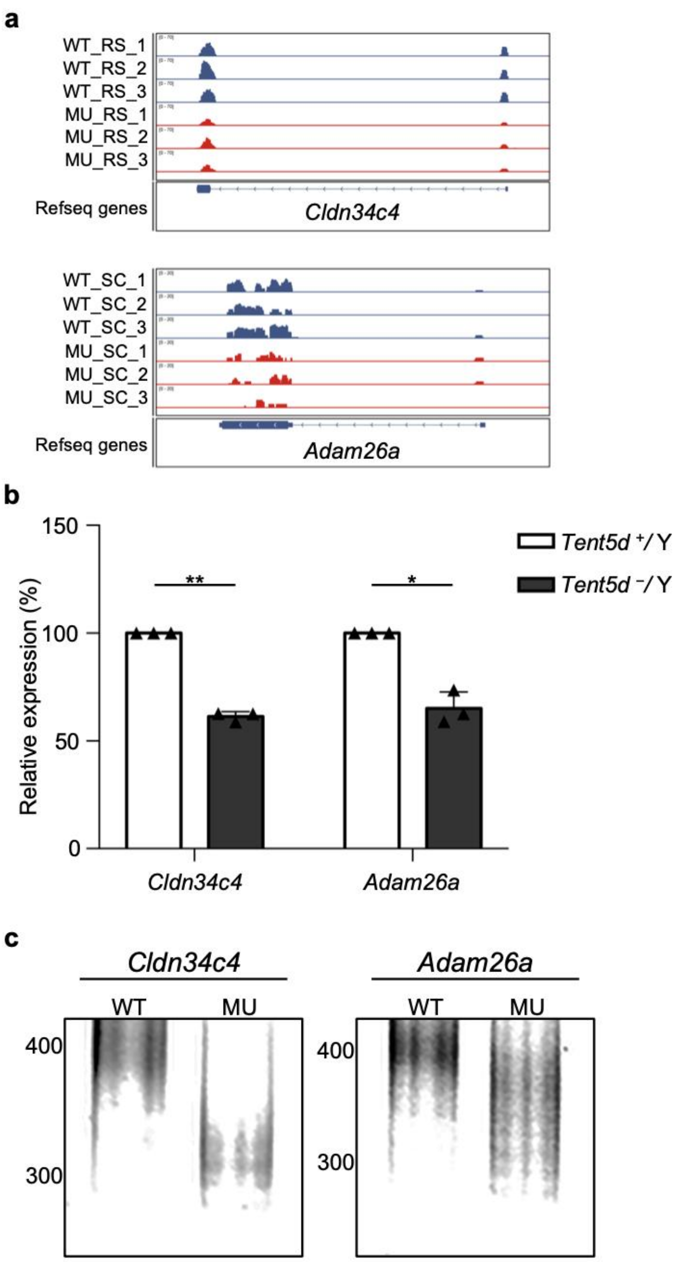

Supplementary Fig. S13 Two cell adhesion-related genes are unstable in *Tent5d*-mutated (MU, *Tent5d*$^{-}$/Y) male germ cells.

a Genome browser snapshot of transcriptome-seq showed that the mRNA levels of *Adam26a* and *Cldn34c4* were downregulated in the spermatocytes and round spermatids, respectively, in *Tent5d*-mutated male mice. **b** RT-qPCR analyses of *Adam26a* and *Cldn34c4* mRNA expressions in the testes of wild-type (WT, *Tent5d*$^{+}$/Y) and *Tent5d*$^{-}$/Y male mice, with *Gapdh* serving as a normalized reference (*P < 0.05, **P < 0.01). **c** PCR-based poly(A) test of *Adam26a* and *Cldn34c4* mRNAs in the testes of WT and *Tent5d*-mutated male mice.

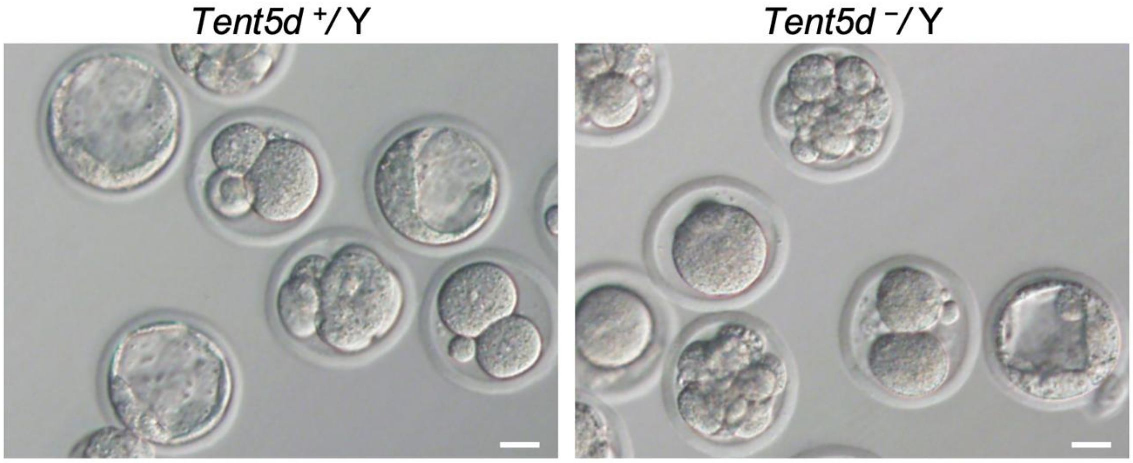

Males providing spermatozoa	No. of oocytes injected	No. of blastocysts	Rate of blastocyst
Tent5d $^{+}$/ Y	15	7	46.7%
Tent5d $^{-}$/ Y	21	1	4.8%

Supplementary Fig. S14 Intracytoplasmic sperm injection (ICSI) is inefficient for overcoming sterility of *Tent5d*-mutated (*Tent5d* $^{-}$/Y) male mice.

Representative blastocysts from ICSI in mice. The rate of blastocysts was dramatically lower in the ICSI group using the spermatozoa from *Tent5d* $^{-}$/Y male mice than that in the control ICSI group using the spermatozoa from wild-type (*Tent5d* $^{+}$/Y) male mice. Scale bars: 25 μm.

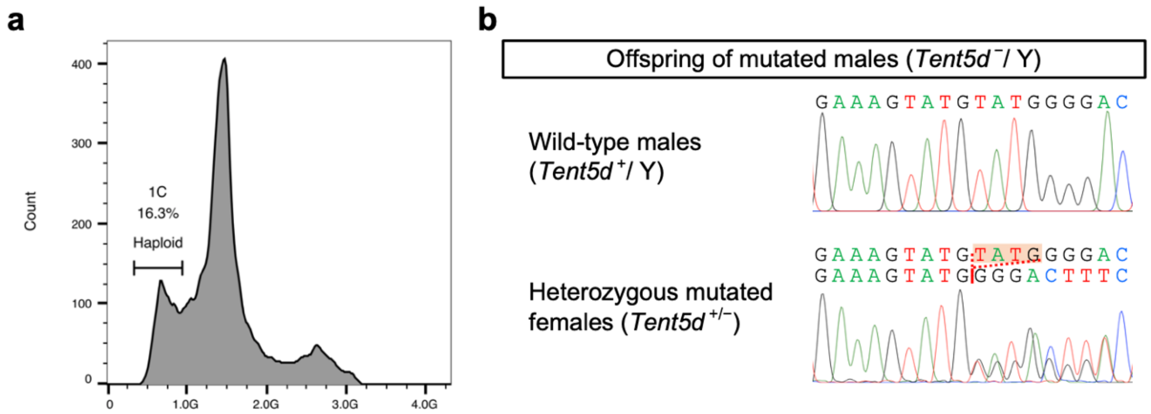

Supplementary Fig. S15 Offspring obtained by intracytoplasmic round spermatid injection (ROSI) using the round spermatids from *Tent5d*-mutated (*Tent5d*$^{-}$/Y) male mice.

a FACS enrichment of round spermatids from the testicular cells of *Tent5d*$^{-}$/Y male mice after Hoechst 33342 staining. **b** Genotyping of the ROSI offspring of *Tent5d*$^{-}$/Y male mice using Sanger sequencing.

Supplementary Tables

Supplementary Table S1. A hemizygous stop-gain variant of *TENT5D* identified in the man affected by oligoasthenoteratozoospermia.

**Variant information**	**Patient HX001 II-1**
cDNA mutation	c.637G>T
Protein alteration	p.Glu213*
Mutation type	Stop-gain
Variant allele	Hemizygous
Inheritance	Maternally inherited
**Allele frequency in human populations**	
1000 Genomes Project	0
All individuals in gnomAD	0
East Asians in gnomAD	0
**Function prediction**	
MutationTaster	Disease causing
CADD	11.8

Note: The NCBI GenBank accession number for *TENT5D* is NM_001170574.2. Variant with CADD value greater than 4 is considered to be deleterious.

Supplementary Table S2. Semen characteristics and sperm morphology in the *TENT5D*-associated case with oligoasthenoteratozoospermia.

	**Patient HX001 II-1**	**Reference**
**Semen parameters**		
Semen volume (mL)	4.0	>1.5[a]
Sperm concentration (10^6/mL)	14.0*	>15.0[a]
Motility (%)	9.0*	>40.0[a]
Progressive Motility (%)	5.0*	>32.0[a]
DNA fragmentation index (%)	55.1*	≤15.0[a]
High DNA stainability (%)	20.2*	≤15.0[a]
**Sperm morphology**		
Acephalic spermatozoa (%)	21.6	≤40.0[a]
Multiple-headed spermatozoa (%)	55.7*	≤2.0[b]

[a]Reference according to the WHO standards[22].

[b]Reference according to the distribution range of morphologically normal spermatozoa observed in 926 fertile individuals[23].

* The asterisks indicate the abnormal values.

**Supplementary Table S3. Off-targets of sgRNA in the mouse genome (GRCm38/mm10).**			
**Location**	**Number of mismatches**	**Sequence (including mismatches shown in red)**	**Genomic location**
chr11:77016828	2	TGGaGaCTGAAAGTATGTATGGG	*Efcab5* intron
chr12:88557377	3	TGaTGtCTGAAtGTATGTATGGG	Intergenic
chr13:48744786	3	TGGTatCTGAAAGTATGTAgTGG	*PTPDC1* intron
chr13:58408464	3	CCCATACATACTTTaAcCCcCCA	*Gkap1* intron
chr13:73432247	3	CCTATcCATACTTcCAGCaACCA	Intergenic
chr14:24745669	3	CCTATACcTACTcTCtGCCACCA	Intergenic
chr15:33621065	3	TGGTGGCTcAAAaTATGTcTAGG	Intergenic
chr18:8029309	3	CCTATACATtCTTTCAGCCttCA	Intergenic
chr5:7088155	2	TGGTGGCTGAAAGTATaTAaAGG	*Zfp804b* intron
chr5:34678407	3	TGGgGGCTGAAAGcATGTtTGGG	Intergenic
chr6:101109614	3	CCTAgACATACTTTCAGCCAggA	Intergenic
chr8:59355251	3	TGGTtGCTGAAAGTATGTtcTGG	*Galntl6* intron
chr8:116323446	3	CCCATcCATcCaTTCAGCCACCA	Intergenic
chrX:18706979	3	CCCAgACATACTTTCtGtCACCA	Intergenic
chrX:97424339	3	TGGTGGCTGtAAGcATaTATAGG	Intergenic
chrX:100984842	3	TGGTGGCaGAAAGgATtTATAGG	*Nhsl2* intron
chrX:106914524	0	TGGTGGCTGAAAGTATGTATGGG	On-target *Tent5d* exon
chrX:162654900	3	TGGTaGCaGAAAGaATGTATTGG	Intergenic

Supplementary Tables S4 and S5 are attached as separate files.

Supplementary Table S6. Clinical outcomes of ICSI cycles using the spermatozoa from the man harboring a hemizygous *TENT5D* stop-gain variant.

**Subject**	**Patient HX001 II-1**	
Age (year)	31	
Age of the wife (year)	32	
Number of ICSI cycles	**Cycle 1**	**Cycle 2**
Number of eggs obtained	18	15
Number of oocytes injected	12	14
Number (and rate) of fertilized oocytes	3 (25%)	3 (21.4%)
Number (and rate) of cleavage embryos	3 (100%)	3 (100%)
Number (and rate) of 8-cells	0 (0%)	0 (0%)

Supplementary Table S7. Primers used for RT-PCR and RT-qPCR assays.		
**Primer name**	**Primer sequence (5'-3')**	**Tm**
H-*TENT5D*-F	TACAGAAATTGGTCAAGGTT	50℃
H-*TENT5D*-R	TGAAATGGTTGTGGAGGT	
M-*Tent5d*-F	GACTTCTTGCCAAAAGGTGTT	55℃
M-*Tent5d*-R	CCAATGAAATGGTTGTGGAGG	
M-*Tent5d*-F-rtpcr	ACATACTCGCAAGCCATA	49℃
M-*Tent5d*-R-rtpcr	AGCCACCACAACAGGA	
M-*Gapdh*-F-rtpcr	GGTGAAGGTCGGTGTGAACG	57℃
M-*Gapdh*-R-rtpcr	CTCGCTCCTGGAAGATGGTG	
M-*Tent5d*-F-rtqpcr	TGGTGGCTGAAAGTATGTATGGG	61℃
M-*Tent5d*-R-rtqpcr	GGTTTGAAGTCACGAACCAACA	
M-*Clu*-F-rtqpcr	CATTCTCCGGCATTCTCTGGG	59℃
M-*Clu*-R-rtqpcr	CTCCCTTGAGTGGACAGTTCTTG	
M-*Cst9*-F-rtqpcr	GTTCCTGGAGGGAGAAGGTAAA	57℃
M-*Cst9*-R-rtqpcr	ACCGACAGTAAACAGGCAGG	
M-*Cst12*-F-rtqpcr	CAGGAGAGAGAAAGGTACGCTG	58℃
M-*Cst12*-R-rtqpcr	GAATGACCTCTGCCCCACTT	
M-*Defb19*-F-rtqpcr	AACTGGTCGTGTCTGGCAAAAA	55℃
M-*Defb19*-R-rtqpcr	AGGCAGCACATCTGGAAAGTT	
M-*Cldn34c4*-F-rtqpcr	GAAGGGACACTTGGACACAGG	58℃
M-*Cldn34c4*-R-rtqpcr	TGGCATCAAATATGTGGAAGCAGC	
M-*Adam26a*-F-rtqpcr	CTCCATTGCCTTTATCTCACCCA	55℃
M-*Adam26a*-R-rtqpcr	TTCAGGTAAGATGTTTTTCACCCA	
M-*Gapdh*-F-rtqpcr	TCATCATCTCCGCCCCTTCT	58℃
M-*Gapdh*-R-rtqpcr	CTGGGTGGCAGTGATGGCAT	

**Supplementary Table S8. Primers used for PAT assays.**		
**Primer name**	**Primer sequence (5'-3')**	**Tm**
Clu-F gene specific	CTGGGAGGGAATCTCCCAGC	60℃
Clu-R poly(A) start	ACACGTATCGCAAGGCGGCTTTTA	
Cst9-F gene specific	CGTACTTCTCTGCATCCGTGA	54℃
Cst9-R poly(A) start	TTCAGAAATGCAATATTTAATGAGC	
Cst12-F gene specific	AGGTACGCTGCACTTACATTG	56℃
Cst12-R poly(A) start	TGTTTTGTAAGTTGACTTTAATTGTCCTGG	
Defb19-F gene specific	GAACAGGCCTACTTCTACTGCA	55℃
Defb19-R poly(A) start	TCAGATGCATACATTTTAATAAGGGG	
Cldn34c4-F gene specific	CCTGCTTTCTTTACAAATGCC	52℃
Cldn34c4-R poly(A) start	TTTTTAAGGTTATGCATGCTATAAATT	
Adam26a-F gene specific	CCACCTCCATTGCCTTTATCTCAT	55℃
Adam26a-R poly(A) start	TTTTTCAGTAGACCTGAAATTCTGTTTTT	
Anchor primer	GCGAGCTCCGCGGCCGCGTTTTTTTTTTTT	68℃

References

1. Liu, C. *et al.* Bi-allelic Mutations in TTC29 Cause Male Subfertility with Asthenoteratospermia in Humans and Mice. *Am. J. Hum. Genet.* **105**, 1168-1181 (2019).
2. Liu, W. *et al.* Bi-allelic Mutations in TTC21A Induce Asthenoteratospermia in Humans and Mice. *Am. J. Hum. Genet.* **104**, 738-748 (2019).
3. Li, H. & Durbin, R. Fast and accurate long-read alignment with Burrows-Wheeler transform. *Bioinformatics* **26**, 589-595 (2010).
4. Ashburner, M. *et al.* Gene ontology: tool for the unification of biology. The Gene Ontology Consortium. *Nat. Genet.* **25**, 25-29 (2000).
5. Kumar, P., Henikoff, S. & Ng, P. C. Predicting the effects of coding non-synonymous variants on protein function using the SIFT algorithm. *Nat. Protoc.* **4**, 1073-1081 (2009).
6. Adzhubei, I. A. *et al.* A method and server for predicting damaging missense mutations. *Nat. Methods* **7**, 248-249 (2010).
7. Schwarz, J. M., Cooper, D. N., Schuelke, M. & Seelow, D. MutationTaster2: mutation prediction for the deep-sequencing age. *Nat. Methods* **11**, 361-362 (2014).
8. Wang, K., Li, M. & Hakonarson, H. ANNOVAR: functional annotation of genetic variants from high-throughput sequencing data. *Nucleic Acids Res.* **38**, e164 (2010).
9. Wang, L. *et al.* CRISPR-Cas9-mediated genome editing in one blastomere of two-cell embryos reveals a novel Tet3 function in regulating neocortical development. *Cell Res.* **27**, 815-829 (2017).
10. Liu, C. *et al.* Deleterious variants in X-linked CFAP47 induce asthenoteratozoospermia and primary male infertility. *Am. J. Hum. Genet.* **108**, 309-323 (2021).
11. Salles, F. J. & Strickland, S. Analysis of poly(A) tail lengths by PCR: the PAT assay. *Methods Mol. Biol.* **118**, 441-448 (1999).

12 Wang, X. *et al.* LARP7-Mediated U6 snRNA Modification Ensures Splicing Fidelity and Spermatogenesis in Mice. *Mol. Cell* **77**, 999-1013 (2020).

13 Bolger, A. M., Lohse, M. & Usadel, B. Trimmomatic: a flexible trimmer for Illumina sequence data. *Bioinformatics* **30**, 2114-2120 (2014).

14 Cai, Z. *et al.* RIC-seq for global in situ profiling of RNA-RNA spatial interactions. *Nature* **582**, 432-437 (2020).

15 Dobin, A. *et al.* STAR: ultrafast universal RNA-seq aligner. *Bioinformatics* **29**, 15-21 (2013).

16 Ramirez, F. *et al.* deepTools2: a next generation web server for deep-sequencing data analysis. *Nucleic Acids Res.* **44**, W160-165 (2016).

17 Thorvaldsdottir, H., Robinson, J. T. & Mesirov, J. P. Integrative Genomics Viewer (IGV): high-performance genomics data visualization and exploration. *Brief. Bioinform.* **14**, 178-192 (2013).

18 Anders, S., Pyl, P. T. & Huber, W. HTSeq--a Python framework to work with high-throughput sequencing data. *Bioinformatics* **31**, 166-169 (2015).

19 Love, M. I., Huber, W. & Anders, S. Moderated estimation of fold change and dispersion for RNA-seq data with DESeq2. *Genome Biol.* **15**, 550 (2014).

20 Gu, Z., Eils, R. & Schlesner, M. Complex heatmaps reveal patterns and correlations in multidimensional genomic data. *Bioinformatics* **32**, 2847-2849 (2016).

21 Yu, G., Wang, L. G., Han, Y. & He, Q. Y. clusterProfiler: an R package for comparing biological themes among gene clusters. *OMICS* **16**, 284-287 (2012).

22 World Health Organization. *WHO Laboratory Manual for the Examination and Processing of Human Semen, 5th edn.* (Cambridge Univ. Press, 2010).

23 Auger, J., Jouannet, P. & Eustache, F. Another look at human sperm morphology. *Hum. Reprod.* **31**, 10-23 (2016).
